# Cytoskeletal Protein 4.1R Is a Positive Regulator of the FcεRI Signaling and Chemotaxis in Mast Cells

**DOI:** 10.3389/fimmu.2019.03068

**Published:** 2020-01-14

**Authors:** Lubica Draberova, Helena Draberova, Lucie Potuckova, Ivana Halova, Monika Bambouskova, Narla Mohandas, Petr Draber

**Affiliations:** ^1^Department of Signal Transduction, Institute of Molecular Genetics of the Czech Academy of Sciences, Prague, Czechia; ^2^Red Cell Physiology Laboratory, New York Blood Center, New York, NY, United States

**Keywords:** mast cell, 4.1R protein, degranulation, chemotaxis, passive cutaneous anaphylaxis

## Abstract

Protein 4.1R, a member of the 4.1 family, functions as a bridge between cytoskeletal and plasma membrane proteins. It is expressed in T cells, where it binds to a linker for activation of T cell (LAT) family member 1 and inhibits its phosphorylation and downstream signaling events after T cell receptor triggering. The role of the 4.1R protein in cell activation through other immunoreceptors is not known. In this study, we used 4.1R-deficient (4.1R-KO) and 4.1R wild-type (WT) mice and explored the role of the 4.1R protein in the high-affinity IgE receptor (FcεRI) signaling in mast cells. We found that bone marrow mast cells (BMMCs) derived from 4.1R-KO mice showed normal growth *in vitro* and expressed FcεRI and c-KIT at levels comparable to WT cells. However, 4.1R-KO cells exhibited reduced antigen-induced degranulation, calcium response, and secretion of tumor necrosis factor-α. Chemotaxis toward antigen and stem cell factor (SCF) and spreading on fibronectin were also reduced in 4.1R-KO BMMCs, whereas prostaglandin E_2_-mediated chemotaxis was not affected. Antibody-induced aggregation of tetraspanin CD9 inhibited chemotaxis toward antigen in WT but not 4.1R-KO BMMCs, implying a CD9-4.1R protein cross-talk. Further studies documented that in the absence of 4.1R, antigen-mediated phosphorylation of FcεRI β and γ subunits was not affected, but phosphorylation of SYK and subsequent signaling events such as phosphorylation of LAT1, phospholipase Cγ1, phosphatases (SHP1 and SHIP), MAP family kinases (p38, ERK, JNK), STAT5, CBL, and mTOR were reduced. Immunoprecipitation studies showed the presence of both LAT1 and LAT2 (LAT, family member 2) in 4.1R immunocomplexes. The positive regulatory role of 4.1R protein in FcεRI-triggered activation was supported by *in vivo* experiments in which 4.1R-KO mice showed the normal presence of mast cells in the ears and peritoneum, but exhibited impaired passive cutaneous anaphylaxis. The combined data indicate that the 4.1R protein functions as a positive regulator in the early activation events after FcεRI triggering in mast cells.

## Introduction

Cells of the immune system, similarly to other cell types, communicate with their environment via a plethora of surface receptors recognizing various soluble or membrane-bound ligands. Recognition of such ligands initiates intracellular signaling events resulting, depending on the overall cellular context, in the activation or inhibition of various immune effector mechanisms, cell differentiation, and/or chemotaxis. Detailed understanding of the structure-function relationships of the immunoreceptors and other surface receptors of the immune cells and identification of their interaction partners is essential for rational approaches toward the treatment of immune system-related diseases (1). For example, the response of mast cells induced by antigen-mediated aggregation of the high-affinity IgE receptor (FcεRI) is potentiated by simultaneous engagement of receptor tyrosine kinase c-KIT (2–4), or inhibited by simultaneous engagement of the inhibitory Fcγ receptors (5). Furthermore, although it is known that the transmembrane adaptor protein, phosphoprotein associated with glycosphingolipid-enriched membrane microdomains (PAG), is an anchor of C-terminal Src kinase, a potent inhibitor of Src family kinases, our studies with bone marrow mast cells (BMMCs) derived from mice with PAG knockout (KO) showed that PAG enhances FcεRI-induced degranulation and chemotaxis responses, but inhibits c-KIT-induced degranulation (6, 7). Our previous studies (8–12), as well as the results of other investigators (6, 13–18), indicated that FcεRI and c-KIT signaling is regulated not only by PAG, but also by other transmembrane adaptor proteins, linker for activation of T cell family (LAT) member 1 (LAT1) and LAT member 2 (LAT2), also called non-T cell activation linker. Other studies indicated an essential role of actin and tubulin in mast cell signaling (19–25), but the connection between the plasma membrane located adaptor proteins, other signal transduction molecules, and cytoskeletal components is not fully understood.

Protein 4.1R is a member of the 4.1 family that functions as a bridge between transmembrane and cytoskeletal proteins [reviewed in (26–28)]. This protein was initially identified in human red blood cells. Later studies showed that there are four 4.1 protein paralogs, including 4.1 R, which is specific for red blood cells, 4.1N, present in neuronal cells, 4.1G, present in numerous cell types, and 4.1B, found predominantly in the brain (28). In higher animals, 4.1 proteins possess a headpiece domain, a 4.1 protein, ezrin, radixin, moesin (FERM) domain, a FERM adjacent (FA) domain, a spectrin-actin binding (SAB) domain, and a unique C-terminal (CTD) domain. 4.1R occurs in two isoforms with apparent molecular mass of 120–135 kDa and/or 80 kDa. These isoforms are the result of splicing of the headpiece domain.

While the role of 4.1R protein in red cells has been extensively documented (26–28), its function in other cell types is not well-defined. It has been shown that 4.1R isoforms are present in the membrane regions, cytoplasm, and nucleus of nucleated cells (27, 29). It has been described that the membrane region localization of 4.1R is important for regulating the functions of several transmembrane proteins (27, 30), plasma membrane receptors (27, 31), transporters (32), and ion channels (33). Using keratinocytes from 4.1R-deficient mice, Chen et al. (34) showed that the 4.1R protein is a positive regulator of cell adhesion, spreading, migration, and motility. They also found that in 4.1R-deficient keratinocytes, the focal adhesion complexes did not localize properly and that β1 integrin levels were reduced. In T cells, Kang et al. (35) described recruitment of the 4.1R protein into the immunologic synapse after T cell receptor (TCR) triggering, hyperactivation of CD4^+^ T cells derived from 4.1R-deficient mice, and hyperproliferation and increased production of interleukin (IL)-2 and interferon γ in 4.1R-deficient T cells. The hyperactivation resulted from enhanced phosphorylation of LAT1 and its downstream signaling molecule ERK. The authors also documented that the 4.1R protein binds directly to LAT1, and thereby prevents its phosphorylation by ZAP-70 kinase. In line with these findings, the authors found that mice deficient in the 4.1R protein displayed enhanced humoral response after immunization with T cell-dependent antigen (35). Further experiments showed that protein 4.1R could suppress activation of CD4^+^ T cells and thereby prevent progression of pathogenic autoimmunity (36). Recently published data concluded that the 4.1R protein is also a negative regulator of TCR signaling in CD8^+^ cells (37). However, the role of the 4.1R protein in the function of other immunoreceptors is not understood.

In the present study, we used 4.1R-deficient (4.1R-KO) mice and corresponding wild-type (WT) littermates and examined the role of 4.1R protein in the FcεRI activation as reflected by degranulation, calcium response, production of cytokines, and chemotaxis in BMMCs *in vitro*. We also compared tyrosine phosphorylation of the proteins involved in early signaling events after FcεRI triggering in 4.1R-KO and WT BMMCs and the interaction of 4.1R protein with transmembrane adaptor proteins LAT1 and LAT2. Finally, we examined the role of 4.1R protein in passive cutaneous anaphylaxis (PCA), which is initiated by antigen-IgE-mediated aggregation of FcεRI in mast cells. Our data imply that in contrast to activation through the T cell immunoreceptor, activation via FcεRI in mast cells is positively regulated by the 4.1R protein under *in vitro* and *in vivo* conditions.

## Materials and Methods

### Mice and Cells

Generation of 4.1R-KO mice and their backcrossing onto the C57BL/6 background has been described (38). Mice were bred and maintained at the Institute of Molecular Genetics in a specific pathogen-free facility and used in compliance with the Institute guidelines.

BMMCs were derived from stem cells in the femurs and tibias of 6–8-week-old 4.1R-KO mice or their WT littermates. The cells were cultured for 8–12 weeks in RPMI-1640 culture medium supplemented with 10% fetal calf serum, minimum essential medium non-essential amino acids, 0.7 mM sodium pyruvate, 2.5 mM L-glutamine, 12 mM D-glucose, antibiotics (100 U/ml penicillin, 100 μg/ml streptomycin), 71 μM 2-mercaptoethanol, recombinant mouse stem cell factor (SCF; 15 ng/ml, PeproTech EC), and recombinant mouse IL-3 (15 ng/ml, PeproTech EC).

### Antibodies and Reagents

Monoclonal mouse antibodies (mAbs) used in this study were as follows: IgE mAb recognizing 2,4,6-trinitrophenol (TNP; IGEL b4.1 clone) (39), anti-FcεRI β chain (40), anti-LYN (41), anti-SYK (42), anti-LAT2 (NTAL; NAP-07 clone) (13), anti-LAT1 (43), anti-CD9, clone 2H9 (11). Polyclonal rabbit antibodies specific for LAT1, LAT2, and LYN were produced by immunization with the recombinant proteins as previously described (44). A polyclonal antibody specific for IgE was produced by immunization of rabbits with isolated IGEL b4.1. A polyclonal antibody specific for 4.1R protein was produced by immunizing goat with recombinant exon 13 (45). Polyclonal antibodies specific for STAT5 (C-17, sc-835), phospholipase C (PLC) γ1 (1249, sc-81), phospho-PLCγ1^Y783^ (sc-12943), ERK1 (c-16, sc-93), phosho-ERK^Y204^ (sc-7976), CBL (c-15, sc-170), phosho-CBL^Y700^ (sc-16140), p-38 (C-20, sc-535), phosho-p38^Y182^ (sc-7975), JNK1 (FL, sc-571), SHIP1 (M-14, sc-1964), c-KIT (H300, sc-5535) phospho-c-KIT^Y568/570^ (sc-18076-R), and horseradish peroxidase (HRP)-conjugated goat antibody specific for mouse IgG (sc-2005), goat antibody specific for rabbit IgG (sc-2004), or donkey antibody specific for goat IgG (sc-2056) were obtained from Santa Cruz Biotechnology Inc. Antibody specific for mouse phospho-SYK^Y519/520^, which is equivalent to human phospho-SYK^Y525/Y526^ (C87C1, Cat. No. 2711), phospho-STAT5^Y694^ (D47E7 XP) Cat. No. 9351), phospho-SHP1^Y564^ (D11G5, Cat. No. 8849), phospho-SAPK/JNK^T183/Y185^ (G9, Cat. No 9255S), m-TOR (Cat. No 2972S), phospho-mTOR^S2448^ (Cat. No. 2971S), phospho-SHIP1^Y1020^ (Cat. No. 3941S), phospho-Lyn^Y507^ (in mouse LYN^Y508^; Cat. No. 2731), phospho-SFKs^Y416^ (in mouse LYN^Y397^; Cat. No. 2101), and PKARI-α/β (Cat. No. 3927S) were obtained from Cell Signaling. Antibody specific for phospho-PKAα/β^T197^ (Cat. No. 2072588) was purchased from Invitrogen. Anti-mouse β1-integrin specific antibody HM β1-1 (Cat. No. 553837), anti-mouse β1-integrin activated epitope, clone 9EG7 (Cat. No. 553715), and HRP-conjugated anti-phosphotyrosine specific mAb (PY20; Cat. No. 610012) were purchased from Becton, Dickinson and Company (BD) Biosciences. Goat antibody specific for hamster IgG conjugated to Alexa Fluor (AF) 488 (Cat. No. A-21110), mouse IgG (Cat. No. A-11001), and Fura-2 acetoxymethyl ester (Fura-2 AM) were obtained from Life Technologies. Anti-mouse FcεRI α chain-fluorescein isothiocyanate (FITC) conjugate (Cat. No. 11-5898) and anti-mouse c-KIT (CD117)-allophycocyanin (APC) conjugate (Cat. No. 17-1171) were obtained from eBiosciences. Anti-mouse FcεRI α chain antibody-Alexa Fluor® 647 conjugate (Cat. No. 134310), anti-mouse c-KIT antibody-PE/Cy7 conjugate (Cat. No. 105814), and Pacific Blue™ anti-mouse Lineage Cocktail (Cat. No. 133310) were obtained from BioLegend. Polyclonal antibody specific for mouse tumor necrosis factor (TNF)-α (Cat. No. 500-P64) was obtained from PeproTech EC. DayLight^TM^ 488-goat antibody specific for rat Fcγ (Cat. No. 112-485-008) was obtained from Jackson Immuno Research Labs. Bovine serum albumin (BSA) conjugated with TNP (15–25 mol TNP/mol BSA) was produced as previously described (46). Thapsigargin was obtained from Invitrogen. ^45^Ca (specific activity, 773 MBq/mg Ca^2+^) was purchased from the Institute of Isotopes Co., Ltd. (Budapest, Hungary). All other reagents were from Sigma-Aldrich.

### Flow Cytometry Analysis

To determine the surface expression of FcεRI and c-KIT, BMMCs were stained with Alexa Fluor 647-conjugated anti-FcεRI and PE/Cy7-conjugated anti-c-KIT antibodies, respectively. After 30 min incubation on ice, the cells were washed three times with ice-cold phosphate-buffered saline (PBS) and samples were evaluated by flow cytometry using LSRII (BD Bioscience). Median fluorescence intensities were analyzed using FlowJo software (ThreeStar, Ashland). To analyze peritoneal mast cells, mice were injected intraperitoneally with 4 ml of PBS supplemented with 1% BSA. After gentle massaging, the mice were sacrificed and the injected PBS with peritoneal fluid was withdrawn, spun down (400 × g, 5 min), and washed in cold PBS. Peritoneal cells were stained with Alexa Fluor 647-conjugated anti-FcεRI and PE/Cy7-conjugated anti-c-KIT antibody. Double-positive cells were gated from Hoechst negative and lineage negative populations. Samples were evaluated using a BD FACSymphony flow cytometer. To quantify the surface expression of β1-integrin and its activated epitope 9EG7, sensitized cells were activated or not with antigen (TNP-BSA; 0.1 μg/ml; 3 min) and non-sensitized cells were activated with SCF (0.05 μg/ml for 3 min). After activation, the cells were then labeled in the first step on ice with the anti-β1-integrin antibody HM β1-1 or 9EG7 for 30 min, followed by 30-min incubation on ice with the corresponding AF488-conjugated anti-hamster or DayLight™ 488 conjugated anti-rat (Fcγ-specific) secondary antibody, respectively. After 30 min of incubation on ice, the cells were washed in ice-cold PBS and analyzed with an LSRII flow cytometer. FlowJo software was used to determine median fluorescence intensities.

### Cell Activation, Degranulation, and Calcium Response

Forty eight hours before experiment, BMMCs were cultured in medium without SCF, followed by 12–14 h incubation in SCF- and IL-3-free medium supplemented with IgE (1,000 × diluted ascites). IgE-sensitized cells were washed in buffered salt solution (BSS; 20 mM HEPES, pH 7.4, 135 mM NaCl, 5 mM KCl, 1.8 mM CaCl_2_, 5.6 mM glucose, 1 mM MgCl_2_) supplemented with 0.1% BSA. Degranulation assays were performed in flat bottomed 96-well plates. IgE-sensitized cells (0.15 × 10^6^) in 30 μl aliquots were activated by mixing with 30 μl of antigen (TNP-BSA conjugate) at various concentrations (0–0.5 μg/ml). Thapsigargin-activated cells were not sensitized with IgE. The degree of degranulation was based on the activity of β-glucuronidase released into the supernatants; for the assay, we used 4-methylumbelliferyl β-D-glucuronide as a substrate (6). Fluorescence in the wells was determined by an Infinite M200 microtiter plate reader (Tecan) at 355 nm excitation and 460 nm emission filters. For the calcium response assay, cells were loaded with Fura-2 AM (1 ng/ml), used as a cytoplasmic reporter, in 2.5 mM probenecid supplemented BSS-0.1% BSA (47). Free intracellular Ca^2+^ was measured in an INFINITE M200 plate reader in a white polysorp 96-well plate NUNC (Thermo Scientific) with excitation wavelengths at 340 and 380 nm and with constant emission at 510 nm. Extracellular calcium uptake was determined by a procedure previously described (6). Briefly, IgE-sensitized or non-sensitized mast cells (2 × 10^6^) were resuspended in 100 μl BSS-BSA containing 1 mM Ca^2+^ and mixed with 100 μl BSS-BSA containing ^45^Ca^2+^ and various concentrations of antigen or thapsigargin. To separate cells with internalized ^45^Ca^2+^ from free ^45^Ca^2+^ the samples were centrifuged at 1,200 × *g* for 15 min at 4°C through 12% BSA. The cells in the pellets were solubilized with 1% Triton X-100. Radioactivity of the samples was counted using scintillation liquid (EcoLite; ICN Biomedicals) and scintillation counter with QuantaSmart software (PerkinElmer).

### Cell Chemotaxis

Chemotaxis of the cells to various chemoattractants was examined in 24-well transwell chamber (Corning) with polycarbonate filters (8-μm pore size). TNP-specific IgE-sensitized BMMCs or non-sensitized cells (0.3 × 10^6^) in chemotaxis medium (120 μl; RPMI-1640 supplemented with 20 mM HEPES and 1% BSA) were added into the transwell insert. Chemoattractants, antigen (TNP-BSA, 0.1 or 0.25 μg/ml), SCF (0.05 μg/ml), or prostaglandin (PG)E_2_ (0.1 μM), were added to the lower compartments in 0.6 ml of chemotaxis medium and cell migration was assessed as previously described (6). Cells migrating through the polycarbonate filter accumulated in the lower wells during the 8 h incubation period at 37°C and 5% CO_2_, were counted in 50 μl aliquots using an Accuri C6 flow cytometer (BD Biosciences). When we tested the effect of anti-CD9 mAb (clone 2H9, 2 μg/ml), inhibitor H-89 (5 μM), and/or caffeine (0.1 mM), IgE-sensitized BMMCs were pretreated with the drugs for 15 min (37°C, 5% CO_2_) and their chemotactic response toward antigen (TNP-BSA, 0.25 μg/ml in lower chamber) was determined in the Transwell system.

### Spreading Assay

To measure cell spreading on fibronectin, wells of 96-well glass-bottom plates (InVitroSci) were coated with fibronectin in PBS (50 μg/ml). After overnight incubation at 4°C, the wells were washed with PBS, and 3 × 10^5^-5 × 10^5^ cells in BSS-0.1% BSA was added into each well. After 30 min at 37°C, the cells were gently washed, and then stimulated with various activators. Thirty minutes later, the cells were fixed for 30 min with 3% paraformaldehyde in PBS at room temperature for 30 min and the fixed cells were washed with 50 mM glycine in PBS. To stain for filamentous actin, the fixed cells were exposed to L-lysophosphatidylcholine (80 μg/ml) in PBS supplemented with Alexa Fluor-488-phalloidin conjugate diluted 1:100 in PBS. After 1 h incubation, the cells were washed thrice in PBS and their nuclei were stained with Hoechst 33258, and the cells were kept in 100 μl of PBS per well until measured. Samples were examined with the Olympus Scan^R^ system. Image processing and quantification of the cell area were completed and analyzed in CellProfiler software (48).

### Immunoprecipitation and Immunoblotting

For immunoprecipitation experiments, activated or non-activated cells were solubilized in ice-cold lysis buffer containing 25 mM Tris-HCl, pH 8.0, 140 mM NaCl, 2 mM EDTA, 1 mM Na_3_VO_4_, 1 μg/ml aprotinin, 1 μg/ml leupeptin, and 1 mM phenylmethylsulfonyl fluoride and supplemented with 0.2% Triton X-100 (for FcεRI analysis). The same immunoprecipitation buffer, but with 25 mM Tris-HCl, pH 7.5 and supplemented with 1% n-dodecyl-β-D-maltoside and 1% Nonidet P-40, was used for LAT1 and LAT2 analysis, or supplemented with 0.5% Triton X-100 and used for coimmunoprecipitation experiments. After solubilization of the cell by incubation on ice for 30 min, the lysates were centrifuged, and postnuclear supernatants were immunoprecipitated with selected antibodies prebound to UltraLink-immobilized protein A (Pierce, Thermo Scientific). The immunoprecipitated proteins were analyzed by immunoblotting with phosphotyrosine-specific PY20-HRP conjugate or with protein-specific antibodies, followed by HRP-conjugated anti-mouse or anti-rabbit IgG antibodies.

For immunoblotting analysis of other proteins, whole-cell extracts (0.25 μg/ml) were prepared by solubilizing cell pellets in SDS sample buffer, followed by 1 min sonication and boiling. Samples were directly analyzed by SDS-PAGE, followed by immunoblotting with 1st layer phospho-protein-specific or corresponding protein-specific antibodies and the by HRP-conjugated secondary antibodies. HRP signal was detected by Luminescent Image Analyzer LAS-3000 (Fujifilm). The HRP signal was quantified by AIDA software (Raytest, GmbH). The levels of phosphorylated proteins were normalized to the loading controls, run in parallel experiments (6).

### Quantification of TNF-α Cytokine

mRNA of IgE-sensitized BMMCs that were non-activated or activated with antigen was extracted using a TurboCapture 96 mRNA kit (Qiagen). M-MLV reverse transcriptase (Invitrogen) was used according to the manufacturer's instructions to produce single-stranded cDNA. Quantitative reverse transcription-polymerase chain reaction (RT-qPCR), amplification of cDNAs, and TNF-α oligonucleotide primers used were described previously (6). Actin, GAPDH, and ubiquitin were used as reference genes, and the expression levels of TNF-α mRNAs were normalized to the geometric means of the reference genes in non-activated control cells. Real-time PCR amplification was performed in 10-μl reaction volumes in a LighCycler 480 II apparatus (Roche Diagnostics). The amount of TNF-α secreted into the media was determined using the immuno-PCR method with nanoparticles armed with TNF-α-specific antibody and DNA template as previously described (7).

### Passive Cutaneous Anaphylaxis

The PCA assay was performed as described previously (47). Briefly, 4.1R-KO and WT mice were anesthetized and then sensitized by intradermal injection of 20 μl of TNP-specific IgE (50 μg/ml PBS) into the left ear or vehicle alone (PBS) into the right ear. Twenty-four hours later, mice were challenged with an intravenous injection of TNP-BSA (5 mg/kg) in 200 μl of PBS containing 1% Evans blue. Forty minutes later, the mice were sacrificed, and the amount of the Evans blue extravasated in their ears was determined.

### Histology

The ears from 4.1R-KO and WT mice were fixed in 4% formaldehyde and embedded in paraffin. Ear sections (5 μm) were stained for 3 min with 1% toluidine blue dissolved in 1% sodium chloride (pH 2.3). The number of mast cells was counted in a blind manner in 50 randomly selected fields from a minimum of three sections of individual tissues from each mouse. The mast cells in the sections were counted under an optical microscope (Olympus DP 50) at magnification 400×, and the mean of mast cells per one field was calculated.

### Statistical Analysis

**Figure 1:**
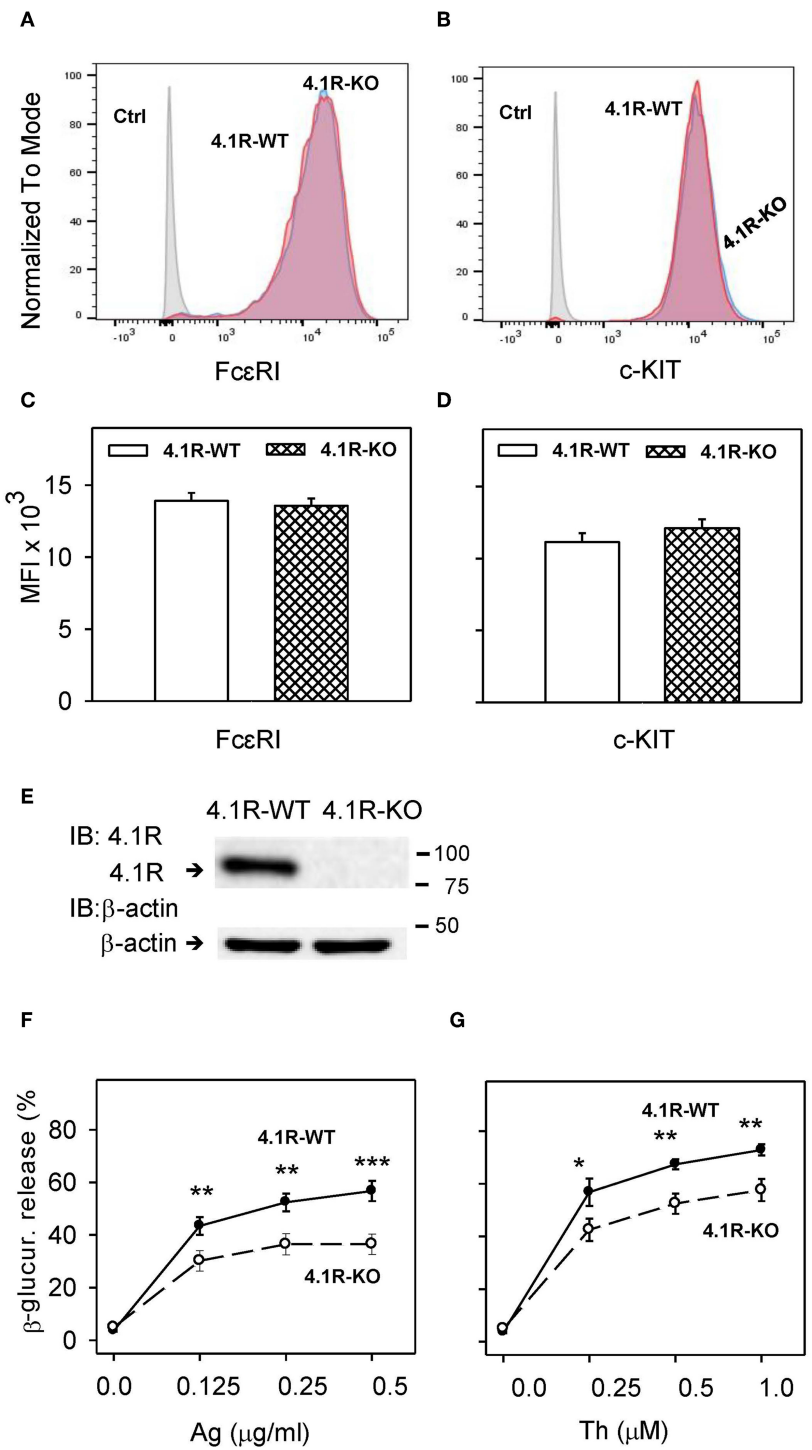
4.1R-deficient BMMCs exhibit normal surface expression of FcεRI and c-KIT, but reduced antigen- and thapsigargin-induced degranulation. **(A–D)** BMMCs derived from 4.1R-WT and 4.1R-KO mice were stained for FcεRI and c-KIT with Alexa Fluor 647-conjugated anti-FcεRI and PE/Cy7-conjugated anti-c-KIT. Unstained 4.1R-WT and 4.1R-KO cells were used as negative controls (Ctrls). Samples were analyzed by flow cytometry. Representative histograms of the expression of FcεRI **(A)** and c-KIT **(B)** gated for live cells after six weeks of culture are shown. **(C,D)** Quantification of representative flow cytometry histograms showing the median fluorescence intensity (MFI) of FcεRI **(C)** and c-KIT **(D)** expressed as means and SEM from three independent experiments. **(E)** 4.1R protein in lysates from 4.1R-WT and 4.1R-KO BMMCs was determined by immunoblotting with the 4.1R-specific antibody. The same membrane was developed for β-actin used as a loading control. Numbers on the right indicate positions of molecular weight markers in kDa. A typical result of four experiments is shown. **(F,G)** Degranulation response of 4.1R-WT and 4.1R-KO BMMCs. The cells were sensitized **(F)** or not **(G)** with TNP-specific IgE (1 μg/ml) and then stimulated for 30 min with various concentrations of antigen (Ag, TNP-BSA; **F**) or thapsigargin (Th; **G**). Data represent means ± SEM calculated from five independent experiments. Statistical significance of differences between 4.1R-WT and 4.1R-KO cells was evaluated with Student's unpaired, two-tailed *t*-test. *P < 0.05; **P < 0.01; and ***P < 0.001.

**Figure 2:**
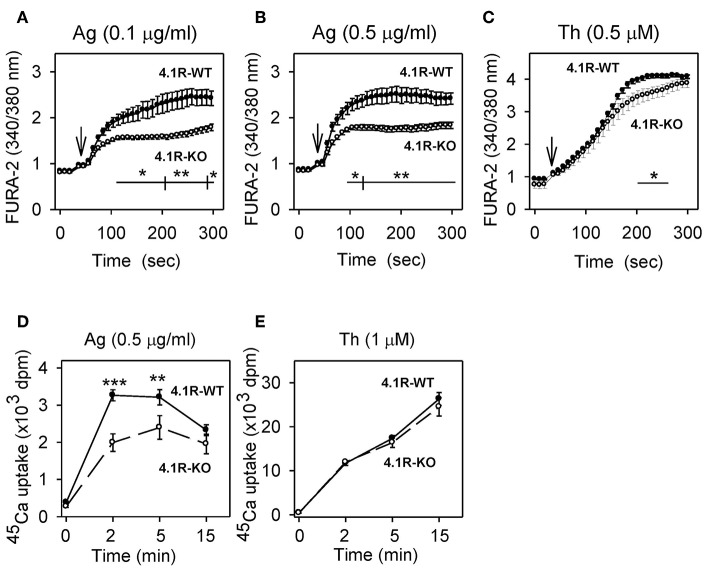
Positive regulatory role of 4.1R protein on antigen-induced calcium response. **(A–C)** Measurement of free intracellular Ca^2+^. 4.1R-WT and 4.1R-KO BMMCs were sensitized with IgE **(A,B)** or not **(C)**, loaded with Fura-2 AM, and then stimulated with antigen (Ag) at a concentration 0.1 μg/ml **(A)** or 0.5 μg/ml **(B)**, or 0.5 μM thapsigargin **(**Th; **C)**, added as indicated by arrows. Free intracellular Ca^2+^ was measured at different time intervals as Fura-2 emission at 510 nm after excitation with 340 and 380 nm. **(D,E)** Measurement of ^45^Ca^2+^ uptake. The cells were sensitized with IgE **(D)** or not **(E)** and then stimulated for various time intervals with antigen at a concentration 0.5 μg/ml **(D)** or 1 μM thapsigargin **(E)** in the presence of 1 mM extracellular ^45^Ca^2+^. The reactions were terminated by centrifugation of the cells through BSA gradient, and cell-bound radioactivity (in cell pellet) was determined. Data represent means ± SEM from 4 to 6 independent experiments performed in duplicates or triplicates. Statistical significance of differences between 4.1R-WT and 4.1R-KO cells was determined using two-way ANOVA with Bonferroni post-test **(A–C)** or unpaired two-tailed Student's *t*-test **(D,E)**. *P < 0.05; **P < 0.01; and ***P < 0.001.

**Figure 3:**
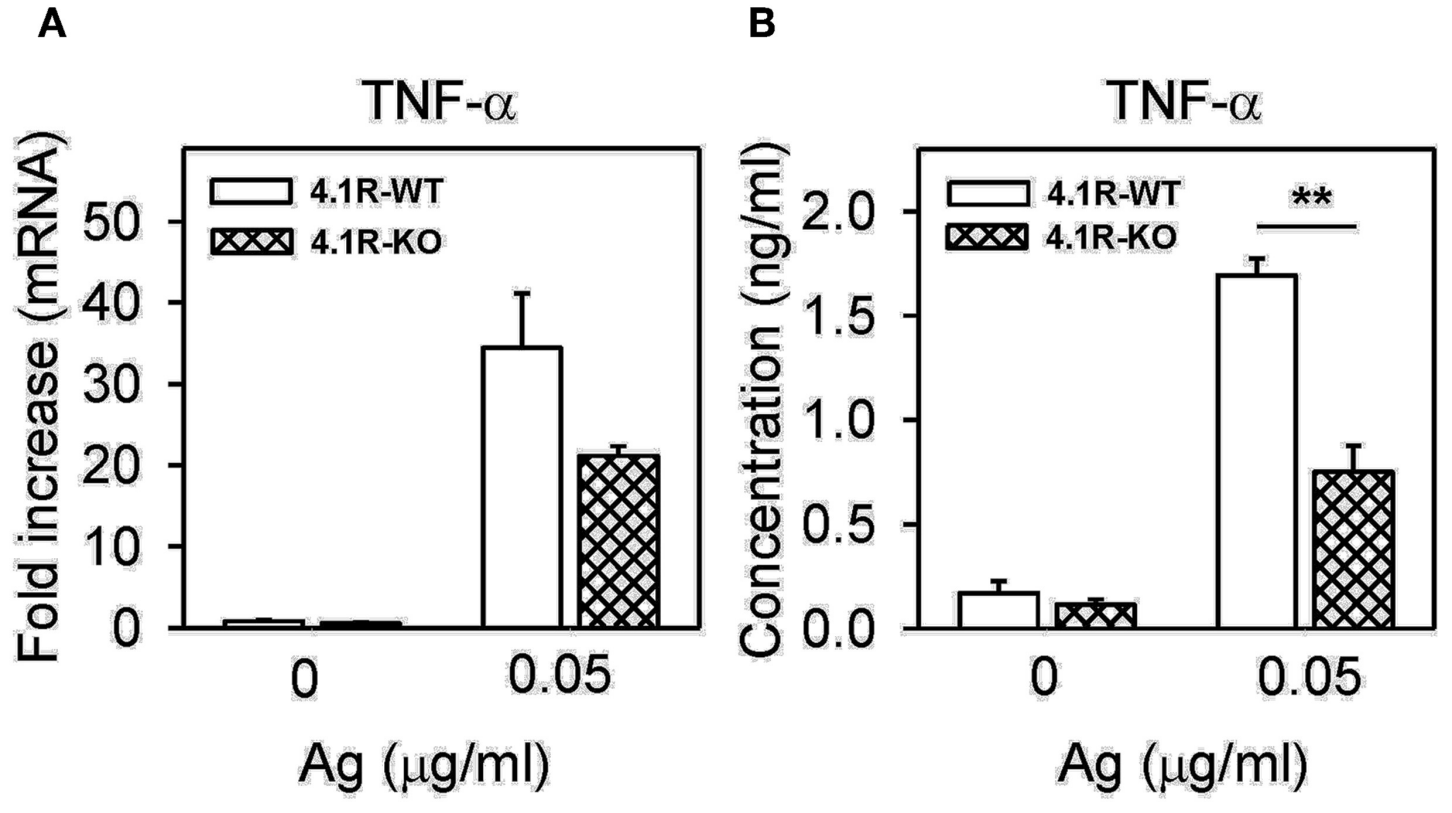
Positive regulatory role of 4.1R protein on TNF-α cytokine gene expression and TNF-α production in FcεRI-activated cells. 4.1R-WT and 4.1R-KO BMMCs were sensitized with IgE and then activated or not with antigen. After 1 h, TNF-α mRNA was quantified by RT-qPCR **(A)**, and after 6 h, TNF-α cytokine secreted into the media was quantified by the immuno-PCR method **(B)**. Means ± SEM were calculated from three independent experiments performed in duplicates or triplicates. Statistical significance of intergroup differences was determined by unpaired two-tailed Student's *t*-test. **P < 0.01.

**Figure 4:**
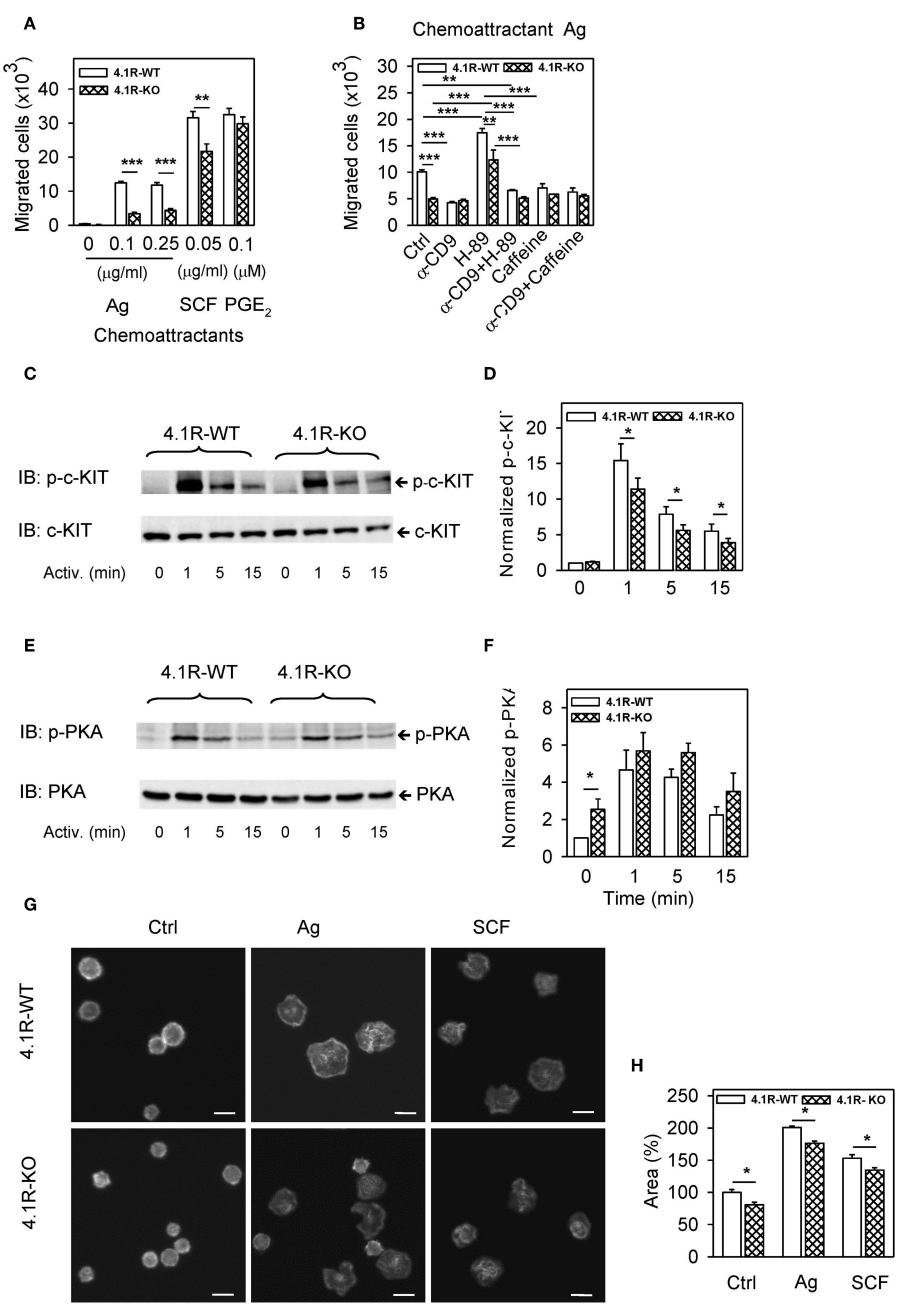
Positive regulatory role of 4.1R on antigen- and SCF-mediated chemotaxis and spreading and cross-talk of 4.1R with tetraspanin CD9 and PKA in chemotaxis toward antigen. **(A)** 4.1R-WT and 4.1R-KO BMMCs were sensitized with IgE and their migration toward the antigen (Ag; TNP-BSA, 0.1 or 0.25 μg/ml in the bottom well) was determined. Migration of IgE non-sensitized cells toward SCF (0.05 μg/ml) and PGE_2_ (0.1 μM) was also determined. Means ± SEMs were calculated from 12 independent experiments performed in duplicates. **(B)** IgE-sensitized 4.1R-WT and 4.1R-KO BMMCs were treated for 15 min at 37°C with anti (α)-CD9 (2 μg/ml), PKA inhibitor H-89 (5 μM), PKA enhancer caffeine (0.1 mM), alone or in combinations, and their effect on chemotaxis toward antigen (0.25 μg/ml in the bottom well) was determined. Cells incubated in chemotaxis medium alone were used as positive controls (Ctrl). **(C–F)** Phosphorylation of c-KIT in cells activated with SCF (0.05 μg/ml; **C,D**) or phosphorylation of PKA in IgE-sensitized cells activated with antigen (0.25 μg/ml, **E,F**). Cells activated for the indicated time intervals were lysed in 2-mercaptoethanol-containing sample buffer, size-fractionated by SDS-PAGE, and analyzed by immunoblotting with antibodies specific for p-c-KIT and loading control c-KIT **(C,D)**, and with p-PKA- and loading control PKA-specific antibodies **(E,F)**. **(C,E)** Show representative immunoblots. **(D,F)** Show the results of densitometry analysis of the corresponding immunoblots in which signals from tyrosine-phosphorylated proteins in activated cells were normalized to the tyrosine-phosphorylated proteins in non-activated 4.1R-WT cells and the amounts of corresponding loading control proteins. Means ± SEM were calculated from a minimum of five independent experiments in each group. **(G)** IgE-sensitized 4.1R-WT and 4.1R-KO BMMCs were attached to the fibronectin-coated glass surface and then non-stimulated (Ctrl) or stimulated with antigen (Ag; 0.25 μg/ml), or SCF (0.05 μg/ml). After 30 min, the cells were fixed and stained for actin with Alexa Fluor 488-phalloidin conjugate. The images are confocal micrographs taken at equatorial planes. Scale bar, 10 μm. **(H)** Cell areas from micrographs as in **(G)** were determined using automated CellProfiller software and the data were normalized to non-activated 4.1R-WT cells. At least 200 cells were evaluated in each experiment. Means + SEM were calculated from three independent experiments. Statistical significance of intergroup differences in **(A,D,F,H)** was determined by unpaired two-tailed Student's *t*-test, and in **(B)** by one-way ANOVA with Tukey's post-test. *P < 0.05; **P < 0.01; and ***P < 0.001.

**Figure 5:**
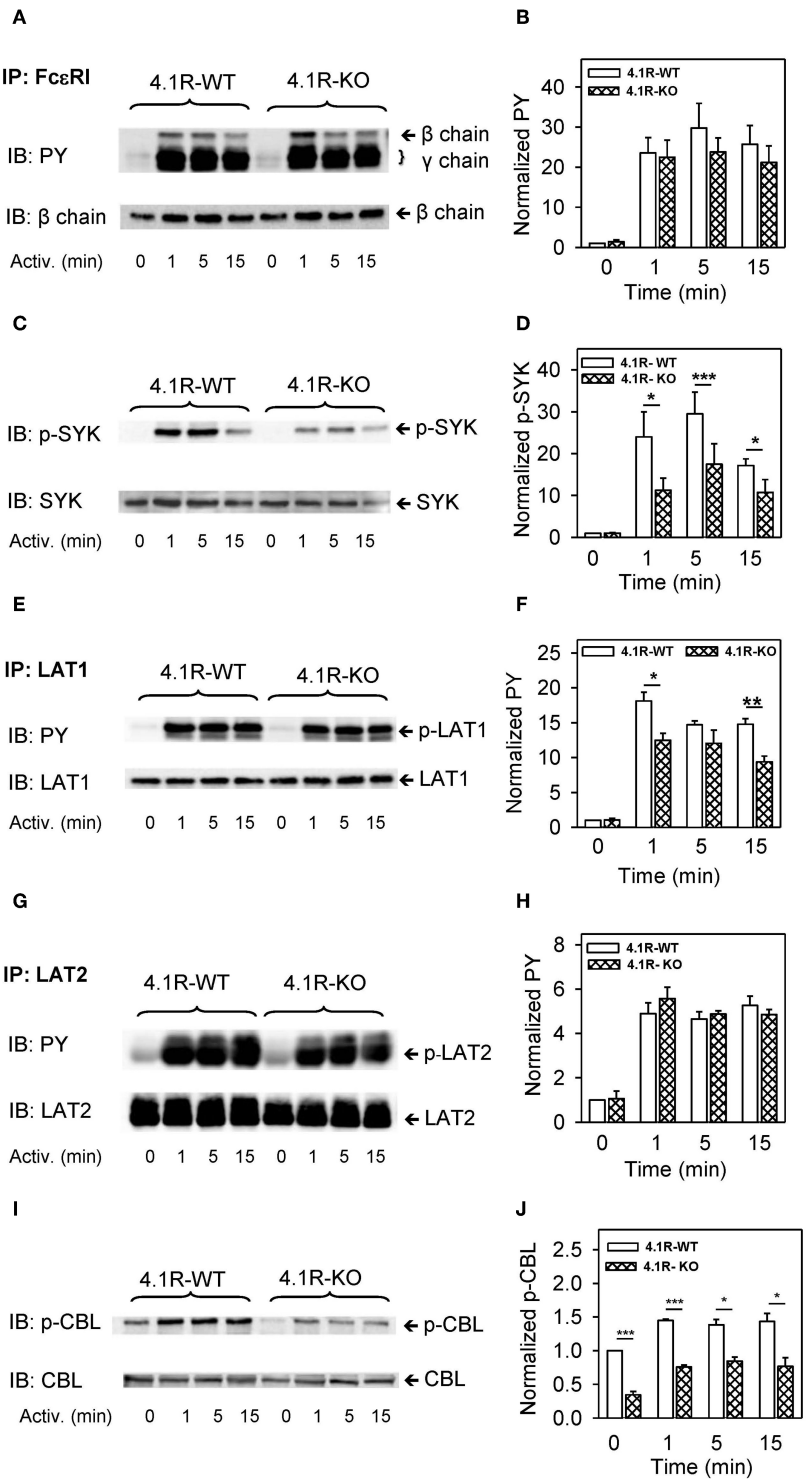
Positive regulatory role of 4.1R on tyrosine phosphorylation of SYK, LAT1, and CBL, but not FcεRI and LAT2, in antigen-activated BMMCs. 4.1R-WT and 4.1R-KO BMMCs were sensitized with IgE and activated for the indicated time intervals with antigen (TNP-BSA; 0.25 μg/ml). The cells were lysed and FcεRI **(A,B)**, LAT1 **(E,F)**, and LAT2 **(G,H)** were immunoprecipitated with the corresponding protein-specific antibodies, followed by size fractionation and examination by immunoblotting with tyrosine-specific mAb PY20-HRP conjugate (PY). For SYK **(C,D)** and CBL **(I,J)**, size-fractionated proteins in cell lysates were directly analyzed by immunoblotting with the antibodies specific for phospho-SYK^Y519/Y520^ and phospho-CBL^Y700^, respectively. For loading controls, the membranes were analyzed by immunoblotting with the corresponding protein-specific antibodies. **(A,C,E,G,I)** Show representative immunoblots from at least five experiments. **(B,D,F,H,J)** Show the results of densitometry analysis of the corresponding immunoblots in which signals from tyrosine-phosphorylated proteins in activated cells are normalized to the tyrosine-phosphorylated proteins in non-activated cells and the amounts of corresponding loading control proteins. Means ± SEM were calculated from a minimum of five independent experiments (three BMMC isolates) in each group. The statistical significance of the difference between 4.1R-WT and 4.1R-KO BMMCs was determined by two-tailed Student's *t*-test. *P < 0.05; **P < 0.01; and ***P < 0.001.

**Figure 6:**
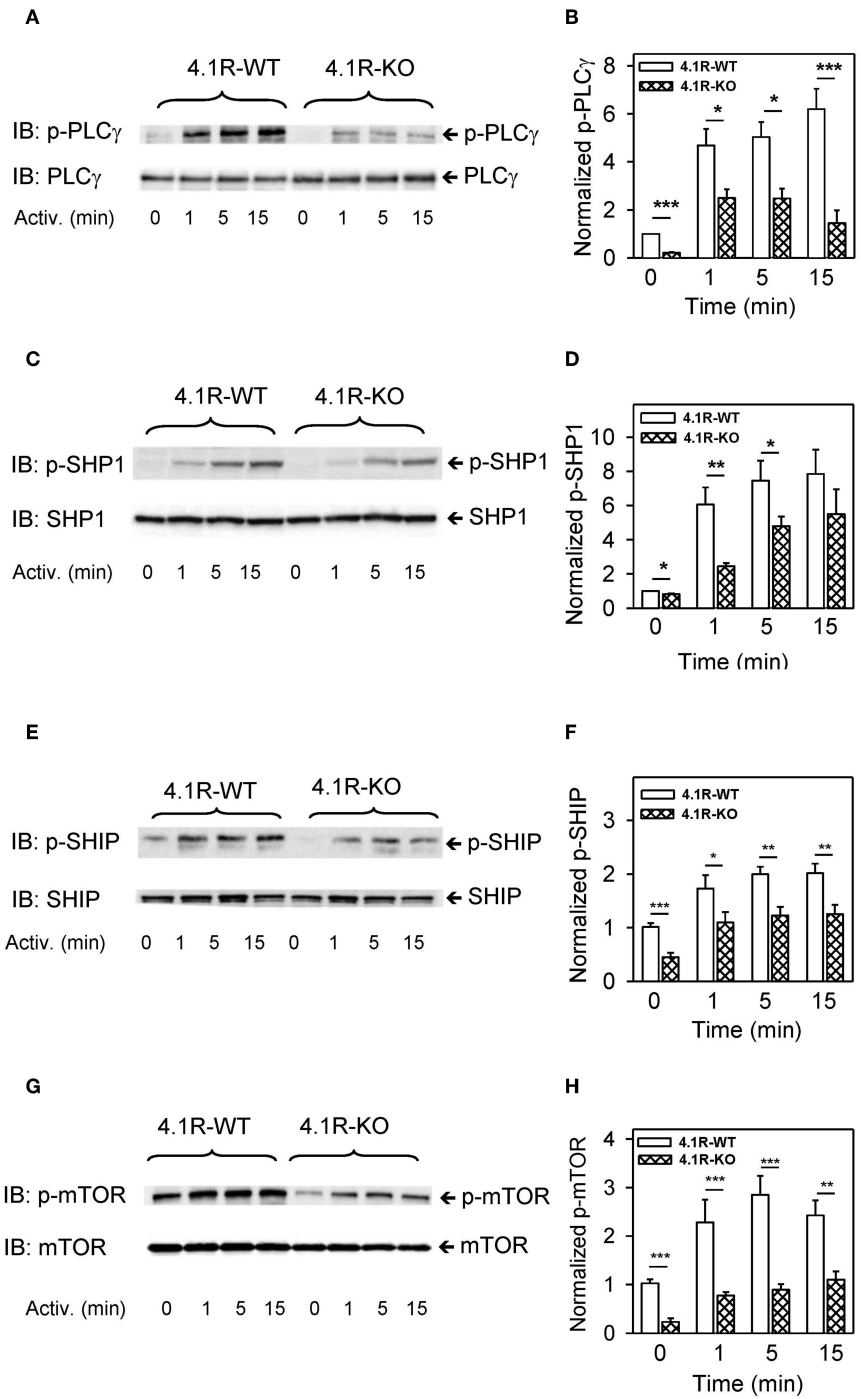
Positive regulatory role of 4.1R in phosphorylation of PLCγ, phosphatases SHP1 and SHIP, and mTOR in antigen-activated BMMCs. 4.1R-WT and 4.1R-KO BMMCs were sensitized with IgE and activated for the indicated time intervals with antigen (TNP-BSA; 0.25 μg/ml). The cells were lysed in 2-mercaptoethanol containing sample buffer, sonicated, size-fractionated by SDS-PAGE, and analyzed by immunoblotting with the antibodies specific for phospho-PLCγ1^Y783^, phospho-SHP1^Y564^, phospho-SHIP1^Y1020^, and phospho-mTOR^S2448^. For loading controls, the membranes were analyzed by immunoblotting with the corresponding protein-specific antibodies. **(A,C,E,G)** Show representative immunoblots. **(B,D,F,H)** Show the results of densitometry analysis of the corresponding immunoblots in which signals from tyrosine-phosphorylated proteins in activated cells were normalized to the tyrosine-phosphorylated proteins in non-activated cells and the amounts of corresponding loading control proteins. Means ± SEM were calculated from a minimum of five independent experiments in each group. The statistical significance of the difference between 4.1R-WT and 4.1R-KO BMMCs was determined by two-tailed Student's *t*-test. *P < 0.05; **P < 0.01; and ***P < 0.001.

**Figure 7:**
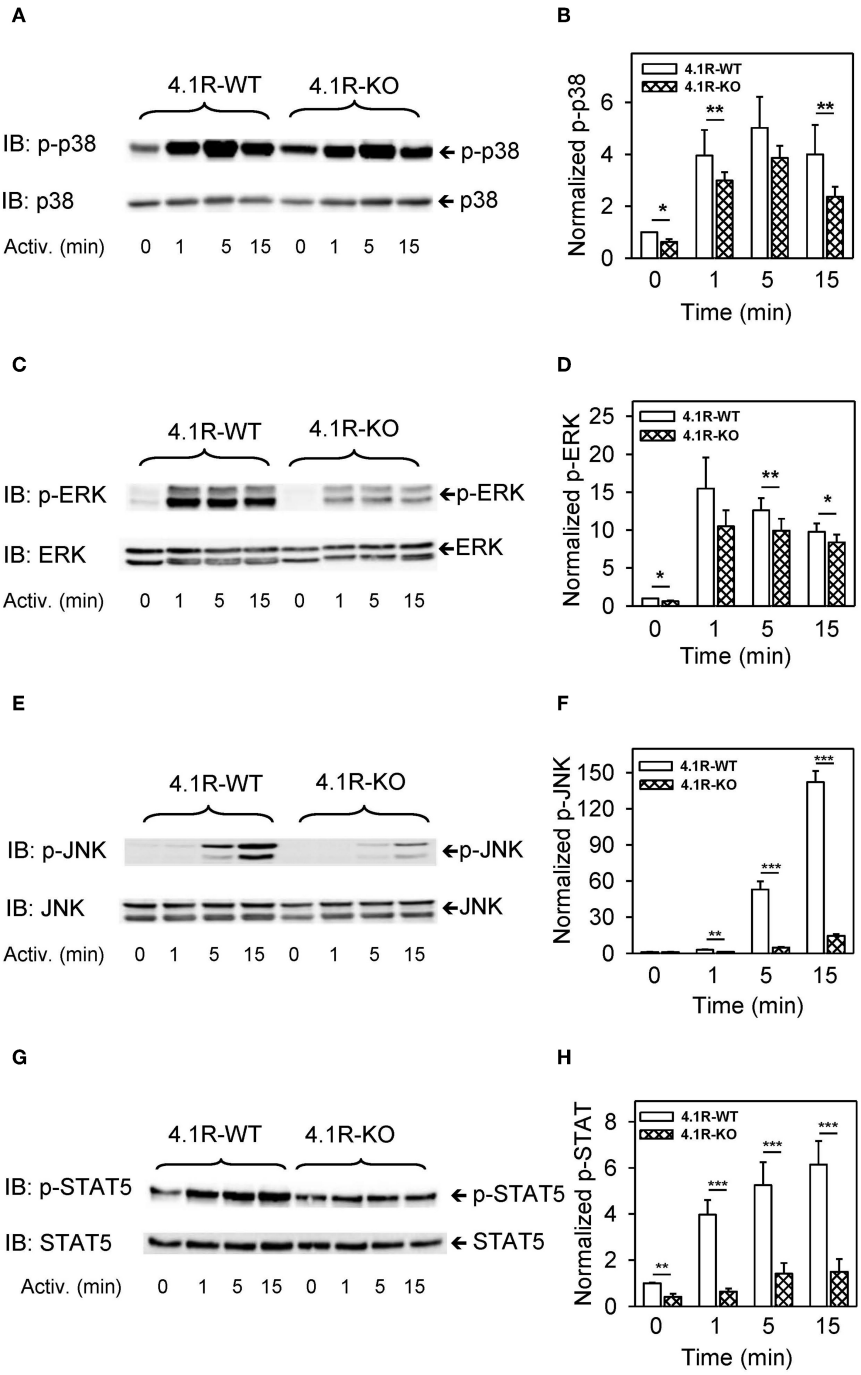
Positive regulatory role of 4.1R in phosphorylation of transcriptional regulators p-38, ERK, JNK, and STAT5 in antigen-activated BMMCs. 4.1R-WT and 4.1R-KO BMMCs were sensitized with IgE and activated for the indicated time intervals with antigen (TNP-BSA; 0.25 μg/ml). The cells were sonicated, size fractionated, and analyzed by immunoblotting with the antibodies specific for phosho-p38^Y182^, phosho-ERK^Y204^, phospho-JNK^T183/Y185^, and phospho-STAT5^Y694^. For loading controls, the membranes were analyzed by immunoblotting with the corresponding protein-specific antibodies. **(A,C,E,G)** Show representative immunoblots. **(B,D,F,H)** Show the results of densitometry analysis of the corresponding immunoblots in which signals from tyrosine-phosphorylated proteins in activated cells were normalized to the tyrosine-phosphorylated proteins in non-activated cells and the amounts of corresponding loading control proteins. Means ± SEM were calculated from a minimum of four independent experiments in each group. The statistical significance of the difference between 4.1R-WT and 4.1R-KO BMMCs was determined by two-tailed Student's *t*-test. *P < 0.05; **P < 0.01; and ***P < 0.001.

**Figure 8:**
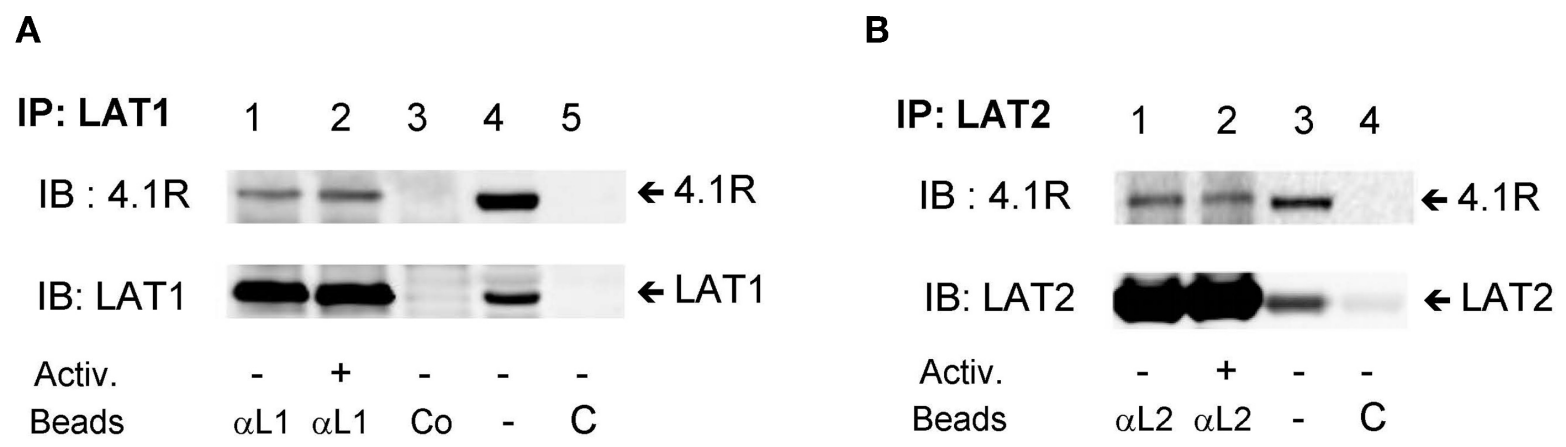
4.1R forms complexes with LAT1 and LAT2 adaptor proteins. BMMCs were sensitized with IgE and activated (+) or not (–) with antigen (TNP-BSA; 0.25 μg/ml) for 5 min. The cells were lysed, and LAT1 **(A)** and LAT2 **(B)** complexes were immunoprecipitated with the LAT1 (αL1) and LAT2 (αL2) specific antibodies immobilized on beads, followed by size fractionation and immunoblotting with 4.1R-specific antibody and anti-LAT1 **(A)** and anti-LAT2 **(B)**. Lysates incubated with the beads armed with normal rabbit serum (**A**, Co, line 3) or beads without antibodies were used as a negative controls (C). As a positive control, whole lysates were directly analyzed by immunoblotting for the presence of 4.1R and LAT1 (**A**, line 4) and LAT2 (**B**, line 3). A typical result from three independent experiments is shown.

Results are expressed as means ± SEM. In experiments where two groups were compared, the statistical significance of intergroup differences was evaluated by unpaired two-tailed Student's *t*-test. In experiments where more than two groups were compared, the statistical significance of intergroup differences was determined by one-way ANOVA with Tukey's post-test using the Prism version 5.04 graphics and statistic software packed (GraphPad Software, LaJolla, CA, USA). Comparison of changes over time between different groups was performed using two-way ANOVA with Bonferroni post-test. ^*^P < 0.05; ^**^P < 0.01; ^***^P < 0.001. The statistical methods and the number of independent experiments used to calculate means ± SEMs are indicated in the corresponding figure legends. In Figures 1–8, experiments based on BMMC cultures derived from different mice are considered as independent.

## Results

### Reduced Degranulation in FcεRI- and Thapsigargin-Activated 4.1R-KO Mast Cells

To determine the role of 4.1R protein in the FcεRI signaling, we isolated bone marrow cells from homozygous F1-descendant, 4.1R-KO and WT mice. The cells were cultured in RPMI-1640 culture medium containing IL-3 and SCF to obtain BMMCs. 4.1R-KO and 4.1R-WT BMMCs exhibited comparable growth properties (not shown), suggesting that the 4.1R protein does not affect the growth response of mast cell progenitors and mast cells to IL-3 and SCF. BMMCs from 4.1R-KO and WT mice expressed comparable amounts of surface FcεRI (Figures 1A,C) and c-KIT (Figures 1B,D). Using anti-4.1R exon 13-specific antibody, we observed one band with the molecular weight of ~80 kDa, corresponding to the 4.1R protein starting from ATG2 (35). As expected, using immunoblot analysis with 4.1R-specific antibody, we found no detectable 4.1R protein in 4.1R-KO BMMCs (Figure 1E).

Mast cell degranulation was examined in 4.1R-KO and WT cells after antigen-IgE complexes-mediated FcεRI aggregation. The extent of degranulation was evaluated by measurement of enzymatic activity of β-glucuronidase released into the extracellular space. The cells were sensitized for 12–14 h with TNP-specific IgE and then activated for 30 min with various concentrations of antigen (TNP-BSA). The data obtained show that when compared to WT cells, 4.1R-KO cells exhibited significantly reduced degranulation at all concentrations of antigen tested (0.125–0.5 μg/ml; Figure 1F). The reduced levels of degranulation did not reflect lower amounts of the cellular β-glucuronidase content as the total levels of β-glucuronidase released from both cell types by Triton X-100 were similar (data not shown). Thus, the absence of 4.1R protein reduces FcεRI-mediated degranulation and does not interfere with the production of β-glucuronidase. Significantly lower degranulation was also observed in 4.1R-KO cells activated with various concentrations of thapsigargin (0.25–1.0 μM; Figure 1G), which induces the release of Ca^2+^ from intracellular stores by inhibiting the endoplasmic reticulum (ER) Ca^2+^-ATPase (49). These data indicate that the 4.1R protein is a positive regulator of FcεRI-induced degranulation in mast cells.

### Reduced Ca^2+^ Response in FcεRI-Activated 4.1R-KO Mast Cells

The early FcεRI-mediated activation event in mast cells involves the release of Ca^2+^ from intracellular stores, followed by the influx of extracellular Ca^2+^ through store-operated Ca^2+^ (SOC) channels in the plasma membrane (50). Ca^2+^ mobilization precedes and is required for mast cell degranulation (51). To determine whether the 4.1R protein has any role in Ca^2+^ signaling, we sensitized 4.1R-KO and WT BMMCs with TNP-specific IgE, loaded them with an intracellular Ca^2+^ indicator, Fura-2 AM, and measured the calcium response after antigen-mediated FcεRI-triggering. We found that Ca^2+^ levels were significantly lower in 4.1R-KO than WT cells when stimulated with both suboptimal (0.1 μg/ml; Figure 2A) and optimal (0.5 μg/ml; Figure 2B) levels of antigen. Thapsigargin-induced Ca^2+^ response was also lower in 4.1R-KO BMMCs than WT cells, even though only during a small window at later stages following triggering (200–250 s; Figure 2C).

Next, we examined whether the 4.1R protein plays a role in the influx of extracellular Ca^2+^. As shown in Figure 2D, stimulation with antigen caused significantly lower uptake of extracellular ^45^Ca^2+^ in 4.1R-KO BMMCs than WT cells, reaching a peak at ~2–5 min after triggering. When the cells were stimulated with thapsigargin, ^45^Ca^2+^ uptake was higher, and there was no significant difference between 4.1R-KO and WT cells (Figure 2E). These data suggest that the reduced levels of antigen-induced degranulation in 4.1R-KO BMMCs are in part attributable to a defect in the signaling pathway leading to the release of Ca^2+^ from intracellular stores.

### Reduced TNF-α Cytokine Gene Expression and Secretion in FcεRI-Activated 4.1R-KO Mast Cells

Aggregation of FcεRI in BMMCs leads to enhanced expression of cytokine genes and secretion of cytokines (52, 53). To determine whether the 4.1R protein has any effect on this process, we examined TNF-α mRNA levels in 4.1R-WT and 4.1R-KO BMMCs activated or not with IgE-antigen complexes. We found that in 4.1R-WT cells, the basal level of TNF-α mRNA in non-activated cells was similar to that in 4.1R-KO cells (Figure 3A). After activation of the cells with antigen for 1 h, 4.1-WT cells exhibited higher levels of TNF-α mRNA than 4.1-KO cells (*P* = 0.09). We also measured secretion of TNF-α into the media. We found that in non-activated cells, there were no significant differences in the basal levels of TNF-α between 4.1R-WT and 4.1R-KO cells. However, in cells activated with antigen for 6 h, the amount of secreted TNF-α was significantly lower in 4.1R-KO cells than in 4.1R-WT cells.

### 4.1R-KO Cells Exhibit Reduced Chemotaxis Toward Antigen and SCF, but Not PGE_2_

An essential aspect of mast cell physiology is chemotaxis toward various chemoattractants (54). Next, we therefore compared chemotaxis toward antigen, SCF, and PGE_2_ in cells differing in the 4.1R protein expression. In the transwell migration assay, 4.1R-KO cells exhibited significantly lower chemotaxis toward antigen and SCF than WT cells (Figure 4A). In contrast, chemotaxis toward PGE_2_ was similar in both cell types (Figure 4A), suggesting that the 4.1R protein is involved in signaling from FcεRI and c-KIT, rather than in the overall capacity of the cells to move toward the chemoattractant.

In our previous study (11) we found that pretreatment of BMMCs with a tetraspanin CD9-specific mAb (2H9) inhibited chemotaxis of IgE-sensitized cells toward antigen. To determine whether 4.1R-KO cells would also be affected by anti-CD9 antibody, we evaluated chemotaxis of both cell types after pretreatment with anti-CD9 mAb. We found that chemotaxis toward antigen was reduced by anti-CD9 pretreatment in WT cells, as expected (Figure 4B). Interestingly, chemotaxis toward antigen was not affected by anti-CD9 in 4.1R-KO cells; both cell types (WT and 4.1R-KO) with bound CD9-specific antibody exhibited comparable chemotaxis toward the antigen. These data suggested that 4.1R could be involved in the cross-talk between CD9 and FcεRI-generated chemotaxis signal.

It has also been described that CD9 is involved in the activation of cAMP/protein kinase A (PKA) (55) and that PKA regulates chemotaxis in various cell types (56, 57). To determine whether there is any difference in the dependence of 4.1R-KO and WT cells on PKA activity, we exposed IgE-sensitized cells to PKA-specific inhibitor, H-89, and measured chemotaxis toward the corresponding antigen. We found that chemotaxis in both 4.1R-KO and WT cells was significantly and comparably increased after H-89 treatment (Figure 4B). This finding suggests that the 4.1R protein is not involved in negative regulation of chemotaxis by PKA. When the cells were pretreated with both H-89 and anti-CD9, chemotaxis was inhibited in both cell types to a similar extent as in the cells exposed to anti-CD9 alone (Figure 4B). The inhibitory role of PKA in chemotaxis of WT cells was supported by experiments in which the cells were pretreated with caffeine, a phosphodiesterase inhibitor, which raises intracellular levels of cAMP and thereby activates PKA (58). Pretreatment of the cells with caffeine alone or in combination with anti-CD9 antibody resulted in inhibition of antigen-mediated chemotaxis in WT cells but not in 4.1R-KO cells, resulting in comparable chemotaxis in both cell types (Figure 4B).

The observed reduction of chemotaxis toward SCF could be partly explained by reduced phosphorylation of c-KIT in 4.1R-KO BMMCs when compared to 4.1R-WT cells (Figures 4C,D). On the other hand, phosphorylation of PKA was significantly enhanced in non-activated 4.1R-KO cells when compared to 4.1R-WT cells (Figures 4E,F). These findings support the data in Figure 4B, suggesting a direct or indirect cross-talk between 4.1R and PKA that could be responsible, at least in part, for the reduced chemotaxis in 4.1R-KO cells.

### 4.1R-KO Cells Exhibit Reduced Spreading on Fibronectin

To investigate the possible involvement of 4.1R protein in adhesion of mast cells to fibronectin, we sensitized 4.1R-KO and WT BMMCs with IgE, transferred them to the fibronectin-coated glass surface and then stimulated them or not with antigen or SCF. After 20 min, the cells were fixed, stained for actin with Alexa Fluor 488-phalloidin conjugate, and confocal microscopy images were obtained (Figure 4G). Detailed analysis of the images using CellProfiller software showed that when compared to non-activated WT cells, 4.1R-KO cells had significantly reduced ability to spread on fibronectin. After exposure to antigen or SCF, both cell types exhibited enhanced cell areas bound to fibronectin, but 4.1R-KO cells exhibited significantly reduced spreading on fibronectin (Figure 4H).

Adhesion and spreading of mast cells on fibronectin is in part dependent on β1-integrin (59), which is present on cell surfaces in both non-activated and activated forms. These two conformations can be distinguished and their expression on the cell surface can be monitored by flow cytometry with antibodies specific for an open (active) conformation (60, 61) and antibodies that bind both forms of β1-integrin. Using an antibody specific for activated β1-integrin, we found that in non-activated cells, there was a small but significantly higher fraction of active β1-integrin in 4.1R-KO cells than in WT cells (Figure S1A). After stimulation with antigen or SCF, the levels of activated β1-integrin were enhanced in both WT and 4.1R-KO cells, but no significant differences between the two cell types were observed (Figure S1A). When the levels of total integrin were examined, no significant differences between non-activated or antigen-activated WT and 4.1R-KO cells were noticed. However, in 4.1R-KO cells activated with SCF, total β1-integrin was lower than in WT cells (Figure S1B). When the ratios between activated and total β1-integrin were analyzed, significantly higher values were observed in non-activated as well as in antigen- and SCF- activated 4.1R-KO cells than in WT cells (Figure S1C). These data suggest that the 4.1R protein in mast cells is involved in the expression of β1-integrin and its active form in mast cells. However, the observed pattern of β1-integrin expression does not explain the lower spreading of 4.1R-KO cells on fibronectin.

### Reduced Antigen-Induced Phosphorylation of Signal Transduction Proteins in 4.1R-KO Cells

Critical molecular events in FcεRI signaling involve phosphorylation of the FcεRI subunits and numerous enzymes and adaptor proteins, which lead to propagation of the activation signal. The first well-defined biochemical step in FcεRI-activated mast cells is Lyn kinase-mediated tyrosine phosphorylation of the FcεRI β and γ subunits (62). To determine whether the 4.1R protein affects this initial activation step, we immunoprecipitated the FcεRI receptor from non-activated and antigen-activated WT and 4.1R-KO cells and analyzed the immunoprecipitates by immunoblotting with phosphotyrosine-specific antibody PY20-HRP conjugate. We found that in non-activated cells, tyrosine phosphorylation of the FcεRI β and γ subunits was weak, and no difference between WT and 4.1R-KO cells was observed. After activation with antigen, rapid phosphorylation of the FcεRI β and γ subunits was observed in WT cells, as expected (62, 63), and again no difference between WT and 4.1-KO cells was noticed (Figures 5A,B). These data indicate that the 4.1R protein is not involved in the earliest biochemical step of FcεRI signaling, tyrosine phosphorylation of the FcεRI β and γ subunits. When FcεRI immunoprecipitates from 4.1R-WT cells were analyzed by immunoblotting for the presence of 4.1R, none was detected (not shown).

A crucial player in early stages of FcεRI-mediated signal transmission is SYK, which binds to the phosphorylated FcεRI γ subunit and actively phosphorylates numerous substrates (64–66). Using an antibody specific for phosphorylated tyrosine within the activation loop of mouse SYK^Y519/Y520^ (corresponding to human SYK^Y525/Y526^), we found that 4.1R-KO cells exhibit reduced phosphorylation of these tyrosines at all time intervals after antigen activation (Figures 5C,D). Significantly reduced tyrosine phosphorylation was also observed in LAT1 transmembrane adaptor protein at 1 and 15 min after activation (Figures 5E,F). Interestingly, tyrosine phosphorylation of another adaptor protein, LAT2, which is similar to LAT1 (67), was not affected by the absence of 4.1R (Figures 5G,H). On the other hand, a multifunctional protein with a ubiquitin E3 ligase activity, CBL (68), showed significantly reduced tyrosine phosphorylation at tyrosine 700 in 4.1R-KO cells (Figures 5I,J). Interestingly, CBL^Y700^ phosphorylation was inhibited not only in FcεRI-activated cells but also in non-activated cells.

Antigen-mediated tyrosine phosphorylation of the FcεRI β and γ subunits, LAT1, and LAT2 are mediated, at least in part, by the activity of LYN kinase (53). The enzymatic activity of LYN is negatively regulated by CSK-mediated phosphorylation of its C-terminal Y^508^ and positively regulated by auto-phosphorylation of its Y^397^. To determine the regulatory role of 4.1R in phosphorylation of these tyrosines, the cells were activated as above and analyzed by immunoblotting with the corresponding phosphotyrosine-specific antibodies. Data presented in Figures S2A–D show that phosphorylation of LYN^Y508^ and LYN^Y397^ is not significantly affected by the absence of 4.1R.

Further analysis showed that phosphorylation of PLCγ1 at tyrosine residue Y783, which is critical for the lipase activation (69, 70), was significantly reduced at all time intervals after FcεRI triggering (Figures 6A,B). Activation of mast cells is also regulated by SHP1 and SHIP phosphatases (71), and both of them exhibited lower tyrosine phosphorylation (Figures 6C–F) in 4.1R-KO than WT BMMCs. In 4.1R-KO cells, we also noticed reduced serine phosphorylation of mTOR^S2448^ (Figures 6G,H), which is associated with mTORC1 signaling (72), a key component of various signaling pathways in mast cells (73).

Our finding that phosphorylation of numerous signaling pathways is inhibited by the absence of 4.1R protein (Figures 5, 6) and reduced expression of cytokine TNF-α gene in activated cells (Figure 3) led us to examine several transcriptional regulators, which are known to exhibit increased phosphorylation in FcεRI-activated cells. We found that phosphorylation of mitogen-activated protein kinases p38^Y182^ (Figures 7A,B), ERK^Y204^ (Figures 7C,D), JNK^T183/Y185^ (Figures 7E,F), and STAT5^Y694^ (Figures 7G,H) exhibited reduced phosphorylation in antigen-activated 4.1R-KO cells when compared to WT cells.

### Interaction of 4.1R Protein With LAT1 and LAT2 Immunocomplexes

In a previous study, Kang et al. (35) found that in T cells, the 4.1R protein interacts with LAT1, and in this way inhibits phosphorylation of LAT1 by ZAP-70 and negatively regulates T cell receptor-mediated signaling. In contrast, our data show that LAT1, but not closely related LAT2 adaptor protein, exhibits lower tyrosine phosphorylation in 4.1R-KO cells (Figures 5E–H). To determine whether the 4.1R protein forms complexes with LAT1 and/or LAT2, we performed immunoprecipitation assays followed by immunoblotting with protein-specific antibodies and found that the 4.1R protein in non-activated cells co-immunoprecipitated with both LAT1 and LAT2 (Figures 8A,B; line 1). No dramatic changes were observed when 4.1R protein was analyzed in LAT1 and LAT2 immunoprecipitates from antigen-activated cells (Figures 8A,B; line 2). Lysates incubated with control beads armed with non-immune serum antibodies or beads without protein-specific antibodies (Figures 8A,B; lines 3 and 5) showed no signal, indicating the specificity of the assay.

### Reduced PCA in 4.1R-KO Mice

**Figure 9:**
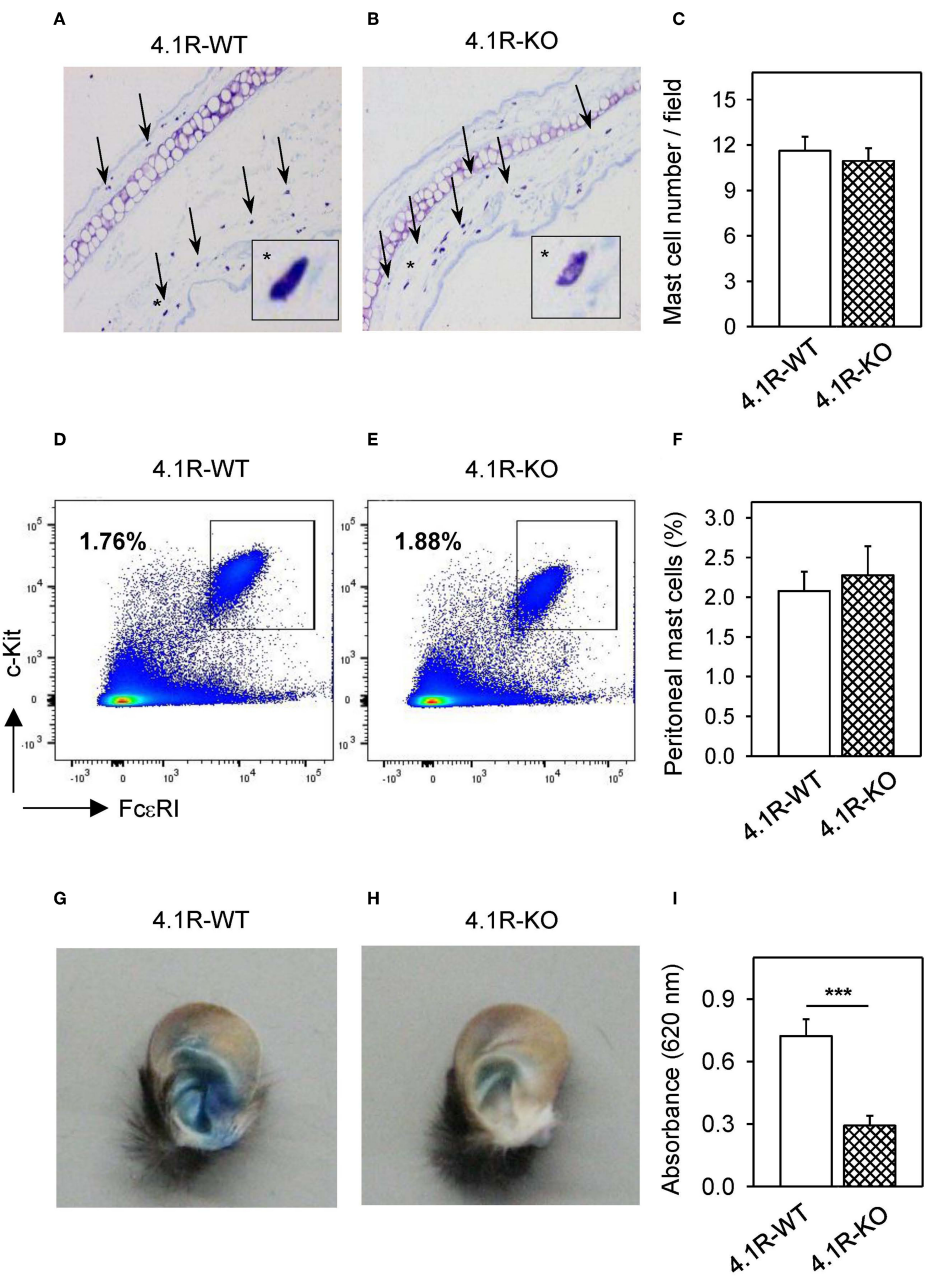
4.1R-KO mice possess normal numbers of mast cells in the ear tissue and peritoneal cavity but exhibit impaired PCA. **(A–C)** The ear tissue from 4.1R-WT mice **(A)** and 4.1R-KO mice **(B)** was fixed with formaldehyde, embedded in paraffin, sectioned, and stained with toluidine blue for histological quantification of mast cells. The representative images were taken at magnification 100x. Arrows and asterisks indicate toluidine blue-positive mast cells. The numbers of mast cells in histological preparations were counted and the data are presented as the means ± SEM of mast cells per microscopy field calculated from 50 fields in each mouse and six mice for each genotype **(C)**. **(D–F)** Peritoneal mast cells from 4.1R-WT mice **(D)** and 4.1R-KO mice **(E)** were stained with FcεRI-specific antibody-Alexa Fluor 647 conjugate and c-KIT-specific antibody-PE/Cy7 conjugate and analyzed by flow cytometry. Means ± SEM of the percentage of mast cells was calculated from the peritoneal cavity of six mice of each genotype **(F)**. **(G–I)** PCA assay was performed as described in Materials and Methods. Examples of the left ear of 4.1R-WT mouse **(G)** and 4.1R-KO mouse **(H)** are shown. **(I)** Quantitative data for ear tissue-extracted Evans blue from IgE-sensitized and antigen-exposed left ear from 4.1R-WT and 4.1R-KO mice. Means ± SEM were calculated from 12 4.1R-WT mice and 11 4.1R-KO mice. Statistical significance of intergroup differences was evaluated by unpaired two-tailed Student's *t*-test **(C,F,G)**. ***P < 0.001.

To determine whether 4.1R protein deficiency has any effect of mast cell activation *in vivo*, we used PCA assays in which local activation of mast cells results in increased vascular permeability and can be visually monitored by leakage of the Evans blue dye in the reaction site. By histological analysis, we first documented that there is no significant difference in the number of mast cells in the ears of 4.1R-KO and WT mice (Figures 9A–C). Similarly, there was no difference in the number of mast cells in the peritoneal cavity of 4.1R-KO and WT mice (Figures 9D–F). For the PCA assay, we sensitized 4.1R-KO and WT mice with TNP-specific IgE mAb injected into the left ears. PBS was injected into the right ears, which served as controls. Twenty-four hours after the challenge with intravenous injection of antigen TNP-BSA in PBS with Evans blue, we noted significantly lower net extravasation of Evans blue in 4.1R-KO mice than in WT mice (Figures 9G–I). These data imply that the 4.1R protein has a positive regulatory role in FcεRI signaling under *in vivo* conditions.

## Discussion

In the present study, we documented that murine mast cells express cytoskeletal protein 4.1R and that it has important regulatory roles in the mast cell activation and chemotaxis. BMMCs derived from 4.1R-KO mice, when compared to BMMCs from WT mice, exhibited reduced degranulation when stimulated by aggregation of FcεRI with IgE-multivalent antigen complexes. The reduced degranulation was associated with changes in the FcεRI signalosome and was not due to reduced expression of the surface FcεRI. Furthermore, while antigen-induced phosphorylation of the FcεRI β and γ subunits was not affected by the absence of 4.1R protein, tyrosine phosphorylation of numerous enzymes, which are essential players in mast cells signaling (74), was significantly reduced, implying that this could account for the reduced degranulation. Interestingly, inhibition of degranulation was also observed in 4.1R KO BMMCs stimulated by thapsigargin, which bypasses the chain of signaling events from IgE-antigen-clustered FcεRI to the activation of PLCγ and IP_3_ production. Thapsigargin inhibits the sarco-endoplasmic reticulum Ca^2+^ reuptake ATPase (SERCA), causes depletion of Ca^2+^ stores, and triggers store-operated Ca^2+^ entry (SOCE). It is known that thapsigargin requires functional PI3K activity to induce mast cell degranulation, as was demonstrated with PI3K inhibitors (75).

The finding that 4.1R protein deficiency in FcεRI-activated cells resulted in reduced calcium response and that the Ca^2+^ response was more inhibited in cells activated by antigen than in cells activated by thapsigargin implies that multiple biochemical steps are involved in the increased levels of free intracellular Ca^2+^ in FcεRI-activated cells and that the 4.1R protein plays a role in some of these steps. The data showing that 4.1R-KO cells, when compared to WT cells, exhibited lower uptake of ^45^Ca^2+^ after FcεRI-mediated activation, but the same ^45^Ca^2+^ uptake after thapsigargin-mediated activation, suggest that the 4.1R protein is not involved in the function of ion channels regulating the movement of Ca^2+^ from and into the cytoplasm.

The results of experiments showing that IgE-sensitized 4.1R-KO cells exhibited reduced chemotaxis toward antigen and SCF but not toward PGE_2_ suggest that the reduced chemotaxis toward the antigen and SCF is not the result of the general inability of 4.1R-KO cells to move toward the chemoattractants, but is rather the result of reduced effectiveness of the signaling pathways used to generate a chemotactic signal from FcεRI and c-KIT. This is also supported by the finding of enhanced or reduced chemotaxis toward antigen in cells pretreated, respectively, with PKA-specific inhibitor, H-89, or caffeine, a PKA activator. PKA as a negative regulator of mast cell chemotaxis could be involved in the process as suggested by our finding of significantly reduced phosphorylation of PKA in non-activated 4.1R-WT cells. Previously, we reported that chemotaxis of WT-BMMCs toward antigen is reduced by antibody-mediated aggregation of tetraspanin CD9 (11). In the present study, we found that although 4.1R-KO and WT cells express comparable amounts of surface CD9, only WT cells were inhibited by anti-CD9 in their migration toward antigen. Thus, FcεRI-mediated chemotaxis could be in part regulated by the CD9-4.1R cross-talk. Ha et al. (76) reported that in murine macrophages, CD9 regulates secretion of IL-10 and IL-6 via the cAMP/PKA signaling pathway. Our finding that PKA inhibitor H-89 enhanced chemotaxis toward antigen in both 4.1R-KO and WT BMMCs, but the difference between these two cell types was preserved, suggested that there are other molecules involved in chemotaxis and affected by 4.1R. When the cells were pretreated with caffeine, which activates PKA (58), significant inhibition of chemotaxis toward antigen was observed in WT cells, as expected, but not in 4.1R-KO cells, which, however, displayed a low response. Our finding that non-activated 4.1R-KO cells exhibited enhanced tyrosine phosphorylation of PKA could be related to the reduced migration of the cells toward antigen. The combined data suggest that 4.1R is involved in the regulation of activity of PKA, which serves as a negative regulator of chemotaxis toward antigen.

In addition to reduced chemotaxis, IgE-sensitized BMMCs from 4.1R-KO mice also exhibited impaired spreading to fibronectin. These data are in line with experiments with keratinocytes from the skin of 4.1R KO mice, which showed that their adhesion, spreading, migration, and motility were impaired when compared to WT keratinocytes (34). Furthermore, the 4.1R protein in keratinocytes modulated the surface expression of β1-integrin, probably as a direct association of 4.1R and β1-integrin (34). By detailed analysis of total β1-integrin on the surface of mast cells from 4.1R-KO and WT BMMCs we were unable to document significant differences in the expression of total β1-integrin in non-activated cells or antigen-activated cells. When we examined activated β1-integrin, we found its increased levels in non-activated 4.1R-KO cells, but did not find any difference in antigen-activated cells. In SCF-activated cells, we found lower levels of total β1-integrin in 4.1R-KO than in WT cells. These data, together with the increased ratio of activated vs. total β1-integrin in non-activated and activated 4.1R-KO mast cells, suggest that there is a cross-talk between the 4.1R protein and surface β1-integrin expression and activation. This cross-talk, however, does not explain the reduced adhesion of 4.1R-KO cells to fibronectin.

A significant finding of the present study is the documentation that removal of 4.1R protein resulted in impaired phosphorylation of numerous signal-transduction proteins. As already mentioned, while the 4.1R protein did not affect tyrosine phosphorylation of FcεRI β and γ subunits in antigen-activated cells, it prevented SYK tyrosine phosphorylation on residues 519 and 520, which are located in the activation loop of the kinase domain and are essential for SYK function (77). The reduced activity of the SYK kinase could be directly or indirectly responsible for the reduced phosphorylation of transmembrane adaptor protein LAT1 and PLCγ1. LAT1 is phosphorylated by SYK and serves as a membrane anchor for PLCγ1, which is activated by phosphorylation at tyrosine 783 by SYK (78). PLCγ catalyzes hydrolysis of membrane-bound phosphatidylinositol 4,5-bisphosphate leading to the production of diacylglycerol and inositol 1,4,5-trisphosphate (IP_3_), which binds to its receptor in the ER. This binding leads to the release of calcium from the ER into the cytoplasm. Because phosphorylation of tyrosine 783 is required for the activity of PLCγ1, the reduced phosphorylation of this tyrosine in 4.1R-KO cells could explain, in part, the reduced Ca^2+^ and degranulation response in the 4.1R-KO cells.

Important signaling molecules, which are phosphorylated in FcεRI-activated cells, are also phosphatases SHP1, SHIP, CBL, and mTOR (79). All these molecules exhibited reduced phosphorylation in 4.1R-KO cells. Reduced initial activation of SYK could be responsible for reduced phosphorylation of at least some of these molecules and several transcriptional regulators, including p38, ERK, JNK, and STAT5 (80) and could explain the reduced levels of TNF-α in antigen-activated 4.1R BMMCs.

In our previous study, we found that although similar in structure, LAT1 and LAT2 are located in different plasma membrane microdomains in mast cells (8). Our present finding that the absence of 4.1R protein in FcεRI-activated BMMCs leads to reduced phosphorylation of LAT1, without any effect on phosphorylation of LAT2, supports the concept that these two adaptor proteins participate differently in the formation of FcεRI signalosome and exhibit different susceptibility to protein tyrosine kinases and/or phosphatases (18). Immunoprecipitation studies showed that although both adaptors formed complexes with the 4.1R protein in WT cells, the absence of 4.1R protein resulted in reduced tyrosine phosphorylation of LAT1 but not LAT2. Thus, in FcεRI-activated mast cells, the 4.1R protein could contribute to stabilization, enhanced phosphorylation, and optimal performance of LAT1 microdomains.

Importantly, the role of 4.1R in the activation of mast cells that we documented in *in vitro* studies was confirmed in the *in vivo* system using the widely accepted PCA assay system (81). Our finding of impaired PCA performance of 4.1R-KO mice when compared to WT mice suggests that the absence of 4.1R protein significantly reduces the secretory response of ear tissue-resident mast cells.

Previous studies showed that the 4.1R protein functions as a negative regulator of TCR signaling. In T cells, the 4.1R protein exerts its effect by binding to transmembrane adaptor LAT1, and thereby prevents its phosphorylation by ZAP-70. Furthermore, mice deficient in the 4.1R protein exhibit an elevated humoral response to immunization with T cell-dependent antigen and reduced T cell-dependent tumor formation. Several lines of evidence presented in this study indicate that in contrast to T cells, the 4.1R protein functions in mast cells as a positive regulator of the FcεRI signaling *in vitro* and *in vivo*. The observed discrepancy could reflect the different composition of the FcεRI and TCR signalosomes. In mast cells, the FcεRI signalosome possesses two transmembrane adaptor proteins, LAT1 and LAT2, which function, respectively, as positive (81) and negative (8) regulators of FcεRI-mediated signaling. In contrast, T cells possess only one of these adaptors, LAT1 (66). In our previous work, we noticed that in FcεRI-stimulated BMMCs, tyrosine phosphorylation of SYK is increased in LAT2-KO cells and reduced in LAT1-KO cells. Based on these results, we postulated that phosphorylation of SYK is enhanced by interaction with LAT1, but not with LAT2, and that there is a competition for SYK between these two adaptors (8). In the present study, we show that 4.1R interacts with both LAT1 and LAT2 in mouse mast cells and that there are no dramatic changes in this binding after FcεRI triggering. We also show that 4.1R deficiency leads to reduced tyrosine phosphorylation of LAT and SYK and inhibition of all subsequent activation events. The reduced SYK phosphorylation in 4.1R-KO cells could be caused by a shift in interaction of SYK with LAT1 and LAT2 in favor of LAT2, which is a negative regulator of mast cell signaling (8). It should be noted that the TCR and FcεRI receptor signaling mechanisms differ in several other essential aspects, including different roles of PAG (6), C-terminal Src kinase (7), and Gads (Grb2-related adaptor downstream of Shc) (82). All these differences could contribute to the opposite outcome of 4.1R deficiency in T cells and mast cells.

## Data Availability Statement

The datasets generated for this study are available on request to the corresponding authors.

## Ethics Statement

Mice were bred and maintained in specific pathogen-free facility of the Institute of Molecular Genetics and used in compliance with the Institute guidelines. All protocols, including killing mice by decapitation, were reviewed and approved by the Animal Care and Use Committee of the Institute of Molecular Genetics (Permit number 12135/2010-17210) and was in compliance with the EU Directive 2010/63/EU for animal experiments. All efforts were made to minimize the suffering of the mice.

## Author Contributions

LD and PD designed the study and wrote the manuscript. LD performed most of the experiments. HD performed initial degranulation, immunoblotting, and immunoprecipitation experiments. LP performed PCA assays, immuno-PCR methods, histochemistry, and quantification of mast cells in the ear tissue and peritoneum. IH performed flow cytometry analysis of β1-integrin and RT-qPCR analyses of cytokines. MB analyzed cell spreading on fibronectin. NM provided 4.1R KO mice and anti-4.1R-specific antibody. All authors analyzed the data, read, and approved the final version of the manuscript.

### Conflict of Interest

The authors declare that the research was conducted in the absence of any commercial or financial relationships that could be construed as a potential conflict of interest.

## Supplementary Material

Figure S1 Negative regulatory role of 4.1R on the ratio of activated and total surface β1-integrin. **(A–C)** IgE-sensitized 4.1R-WT and 4.1R-KO BMMCs were non-activated (Ctrl) or activated for 3 min with antigen (TNP-BSA, 0.1 μg/ml) or SCF (0.05 μg/ml), and the presence of surface activated β1-integrin **(A)** and total β1-integrin **(B)** was determined by flow cytometry. The ratio between activated and total β1-integrin is also shown **(C)**. The data are normalized to non-activated 4.1R-WT cells. Means ± SEM were calculated from seven independent experiments. Statistical significance of intergroup differences was determined by two-tailed Student's *t*-test. ^*^*P* < 0.05; ^**^*P* < 0.01; and ^***^*P* < 0.001.

Figure S2 Cytoskeletal protein 4.1R is not involved in phosphorylation of regulatory tyrosines of Lyn kinase. 4.1R-WT and 4.1R-KO BMMCs were sensitized with IgE and activated for the indicated time intervals with antigen (TNP-BSA; 0.25 μg/ml). The cells were solubilized, size fractionated, and analyzed by immunoblotting with the antibodies specific for p-LYN^Y508^ **(A,B)** or p-SFKs^Y397^ **(C,D)**. For loading controls, the membranes were analyzed by immunoblotting with the Lyn-specific antibody. **(A,C)** Show representative immunoblots. **(B,D)** Show the results of densitometry analysis of the corresponding immunoblots in which signals from tyrosine-phosphorylated proteins in activated cells were normalized to tyrosine-phosphorylated proteins in non-activated 4.1R-WT cells and the amounts of corresponding loading control proteins. Means ± SEM were calculated from three independent experiments in each group.

## Abbreviations

APC: allophycocyanin
BMMC: bone marrow mast cell
BSA: bovine serum albumin
BSS: buffered salt solution
FcεRI: high-affinity IgE receptor
FERM, 4.1 protein, ezrin, radixin: moesin
FITC: fluorescein isothiocyanate
HRP: horseradish peroxidase
IL: interleukin
IP_3_: 1,4,5-trisphosphate
KO: knockout
LAT: linker for activation of T cells
LAT1: LAT, member 1
LAT2: LAT, member 2
mAb: monoclonal antibody
PAG: phosphoprotein associated with glycosphingolipid-enriched microdomains
PAGE: polyacrylamide gel electrophoresis
PBS: phosphate-buffered saline
PCA: passive cutaneous anaphylaxis
PG: prostaglandin
PKA: protein kinase A
PLC: phospholipase C
RT-qPCR: quantitative reverse transcription polymerase chain reaction
SCF: stem cell factor
SDS: sodium dodecyl sulfate
TCR: T cell receptor
TNF: tumor necrosis factor
TNP: 2,4,6-trinitrophenol
WT: wild-type.
