# Supplementary material for: Cytoskeletal Protein 4.1R Is a Positive Regulator of the FcεRI Signaling and Chemotaxis in Mast Cells
Source: Front Immunol. 2020 Jan 14;10:3068. doi: 10.3389/fimmu.2019.03068 (PMC6970983; doi:10.3389/fimmu.2019.03068)
Supplement: Figure S1 — Negative regulatory role of 4.1R on the ratio of activated and total surface β1-integrin. (A–C) IgE-sensitized 4.1R-WT and 4.1R-KO BMMCs were non-activated (Ctrl) or activated for 3 min with antigen (TNP-BSA, 0.1 μg/ml) or SCF (0.05 μg/ml), and the presence of surface activated β1-integrin (A) and total β1-integrin (B) was determined by flow cytometry. The ratio between activated and total β1-integrin is also shown (C). The data are normalized to non-activated 4.1R-WT cells. Means ± SEM were calculated from seven independent experiments. Statistical significance of intergroup differences was determined by two-tailed Student's t-test. *P < 0.05; **P < 0.01; and ***P < 0.001. [file Data_Sheet_1.pdf]

SUPPLEMENTARY MATERIAL

Cytoskeletal Protein 4.1R Is a Positive Regulator of the FcεRI Signaling and Chemotaxis in Mast Cells

Lubica Draberova*, Helena Draberova, Lucie Potuckova, Ivana Halova, Monika Bambouskova, Narla Mohandas and Petr Draber*

Correspondence: Dr. Lubica Draberova: draberlu@img.cas.cz

Dr. Petr Draber: draberpe@img.cas.cz

Supplementary material contains supplementary Figures S1 and S2. Legends for these figures are present in the main text.

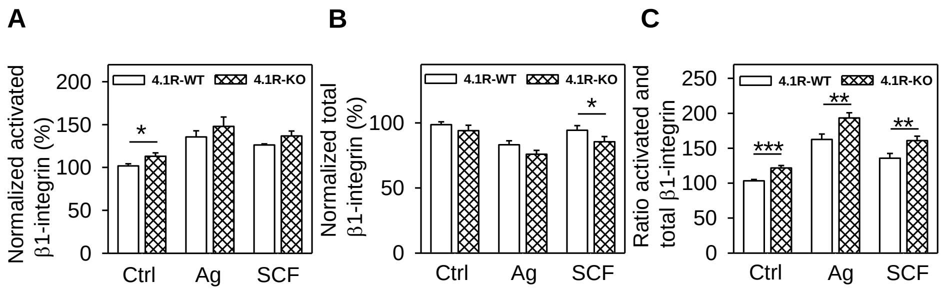
A
B
C
4.1R-WT
4.1R-KO
Normalized activated β1-integrin (%)
Normalized total β1-integrin (%)
Ratio activated and total β1-integrin
Ctrl
Ag
SCF

FIGURE S1.

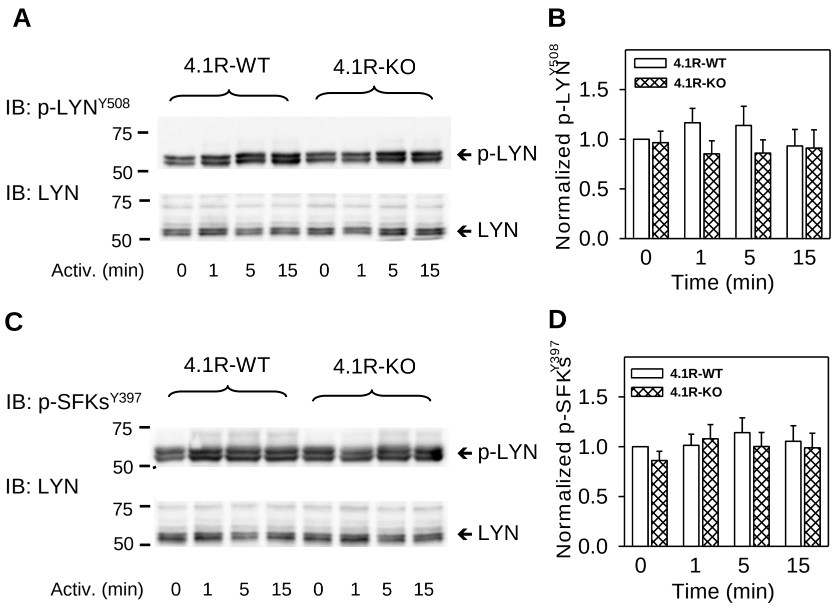
A
4.1R-WT
4.1R-KO
IB: p-LYNY508
75
50
p-LYN
IB: LYN
75
50
LYN
Activ. (min)
0 1 5 15 0 1 5 15
B
4.1R-WT
4.1R-KO
Normalized p-LYNY508
0.0
0.5
1.0
1.5
0 1 5 15
Time (min)
C
4.1R-WT
4.1R-KO
IB: p-SFKsY397
75
50
p-LYN
IB: LYN
75
50
LYN
Activ. (min)
0 1 5 15 0 1 5 15
D
4.1R-WT
4.1R-KO
Normalized p-SFKsY397
0.0
0.5
1.0
1.5
0 1 5 15
Time (min)

FIGURE S2.
